# Probiotic potential of *Enterococcus faecium* SWUN5732 isolated from yak yogurt: integrated *in vitro* and *in vivo* evaluation for yak health

**DOI:** 10.14202/vetworld.2026.1581-1594

**Published:** 2026-04-25

**Authors:** Ming Yang, Xiaobo Li, Longjun Ran, Xuan Ran, Xiaodong Liang, Falong Yang, Dechun Chen, Jian Fu

**Affiliations:** 1 Sichuan Sports College, Chengdu 610000, China; 2 Biosafety Laboratory of West China Hospital of Sichuan University, Chengdu, Sichuan 610041, China; 3 Key Laboratory of Animal Medicine of Sichuan Province, Southwest Minzu University, Chengdu 610041, China; 4 Aba Vocational College, Maoxian 623002, China

**Keywords:** acid tolerance, antibiotic alternative, *Enterococcus faecium*, high-altitude adaptation, intestinal adhesion, mucosal immunity, probiotic potential, yak yogurt

## Abstract

**Background and Aim:**

The increasing emergence of antimicrobial resistance has intensified the search for safe and effective alternatives to antibiotics in livestock production. Probiotics derived from traditional fermented foods represent a promising strategy due to their adaptability and functional diversity. This study aimed to isolate and characterize a plateau-adapted strain of *Enterococcus faecium* from traditional yak yogurt and to comprehensively evaluate its probiotic potential through *in vitro* assays and *in vivo* validation, with a focus on its applicability in yak health management.

**Materials and Methods:**

The strain *E. faecium* SWUN5732 was isolated and identified using morphological characterization and *16S rRNA* gene sequencing. Probiotic properties were assessed through acid (pH 2.0 and 3.0) and bile salt (0.3%) tolerance, hemolytic activity, antibiotic susceptibility, antimicrobial activity against common yak pathogens, and adhesion to Caco-2 cells. Safety evaluation included virulence gene screening targeting *cylA*, *esp*, *asa1*, *gelE*, *agg*, and *efaAfm*, as well as acute oral toxicity testing in mice. Functional efficacy was further examined through a 21-day oral administration study in mice to evaluate body weight gain and intestinal secretory immunoglobulin A levels.

**Results:**

*E. faecium* SWUN5732 demonstrated strong gastrointestinal tolerance, with survival rates of 34.43% at pH 2.0, 60.17% at pH 3.0, and 77.07% in 0.3% bile salts. The strain exhibited no hemolytic activity and was susceptible to clinically relevant antibiotics. It showed broad-spectrum antimicrobial activity against *Escherichia coli*, *Salmonella* spp., and *Staphylococcus aureus*, with inhibition zones ranging from 18 to 37 mm. The adhesion rate to Caco-2 cells was approximately 29%, indicating effective colonization potential. Among the tested virulence determinants, only *efaAfm* was detected, while other genes (*cylA*, *esp*, *asa1*, *gelE*, *agg*) were absent. No acute toxicity or pathological alterations were observed in mice. Notably, oral administration significantly enhanced body weight gain and increased intestinal secretory immunoglobulin A levels (p < 0.01), demonstrating immunomodulatory and growth-promoting effects.

**Conclusion:**

The findings indicate that *E. faecium* SWUN5732 possesses key probiotic attributes, including gastrointestinal resilience, antimicrobial activity, safety, and immunomodulatory capacity. Its origin from high-altitude yak yogurt suggests ecological compatibility with yak production systems. This strain represents a promising candidate as an antibiotic alternative in yak farming; however, further *in vivo* validation in yaks is required to confirm its practical applicability under field conditions.

## INTRODUCTION

The Tibetan population has long practiced the production of yak yogurt using traditional fermentation techniques. This fermented product harbors a diverse microbial community [1], predominantly composed of lactic acid bacteria. The unique geographical and environmental conditions of the plateau play a crucial role in shaping this microbial diversity, enabling the enrichment of lactic acid bacteria with beneficial functional properties, including antioxidant activity [2, 3], antihypertensive effects [4], and prevention of intestinal disorders [5]. Beyond its cultural significance, the microbial diversity of traditional yak yogurt represents a valuable reservoir of novel probiotic strains that are naturally adapted to extreme environmental conditions.

The shift from traditional pastoral systems to intensive livestock farming has introduced new challenges in animal health management. The rapid expansion of intensive farming practices has increased the susceptibility of yaks to recurrent bacterial infections, leading to considerable economic losses in the yak production sector [6, 7]. Consequently, the identification of safe antibacterial alternatives in veterinary medicine has become a major research priority, particularly in response to concerns regarding antibiotic residues and the emergence of drug-resistant pathogens in livestock products. In this context, probiotics have gained increasing attention as effective antibiotic substitutes due to their ability to inhibit environmental pathogens and food spoilage microorganisms [8, 9].

*Enterococcus faecium* is a commensal bacterium present in the gastrointestinal tract of humans and animals and belongs to the lactic acid bacteria group. Previous studies have reported the isolation of probiotic strains of *E. faecium* from various fermented foods, including sausage [10], kimchi [11], and fish products [12]. *E. faecium* exhibits strong adhesion to intestinal epithelial cells [13] and produces bacteriocins, organic acids, and other bioactive compounds that effectively inhibit the growth of pathogenic microorganisms [14]. In poultry production, *E. faecium* has been used to mitigate the adverse effects associated with antibiotic use by modulating the intestinal microbiota [15]. Similarly, dietary supplementation with probiotics containing *E. faecium* has been shown to maintain immune homeostasis in pigs under inflammatory conditions [16].

Despite the recognized importance of probiotics as alternatives to antibiotics in livestock production, research focusing specifically on yak-adapted probiotic strains remains limited. Most existing studies have primarily investigated probiotic applications in conventional livestock such as poultry and swine, with comparatively little attention given to high-altitude ruminants such as yaks [17]. This is a critical limitation because yaks are uniquely adapted to extreme plateau environments characterized by hypoxia, low temperatures, and high ultraviolet radiation, conditions that significantly influence both host physiology and microbial ecology. Consequently, probiotic strains developed for lowland livestock may not exhibit optimal functionality or survival under such harsh environmental conditions.

Traditional fermented yak yogurt represents a rich and largely underexplored source of indigenous microorganisms with potential probiotic properties. However, systematic investigations into the isolation, characterization, and functional validation of these plateau-adapted strains remain scarce. In particular, there is a lack of integrated studies combining *in vitro* probiotic characterization with *in vivo* validation to assess safety, antimicrobial efficacy, intestinal adhesion, and immunomodulatory potential. Furthermore, limited information is available regarding the ability of these strains to combat yak-specific pathogens or enhance host immune responses under physiological conditions. This knowledge gap restricts the development of targeted microecological interventions tailored to yak production systems and hinders progress toward reducing antibiotic dependence in high-altitude livestock farming.

Therefore, the present study was designed to isolate and characterize a plateau-adapted strain of *Enterococcus faecium* from traditional Tibetan yak yogurt and to comprehensively evaluate its probiotic potential. The study aimed to assess key functional properties, including tolerance to acidic and bile salt conditions, antimicrobial activity against common yak pathogens, adhesion capacity to intestinal epithelial cells, and safety through hemolytic activity, antibiotic susceptibility profiling, and virulence gene screening.

In addition, the study sought to validate the biological efficacy of the isolated strain through *in vivo* experimentation, focusing on its effects on growth performance and intestinal mucosal immunity, as indicated by secretory immunoglobulin A levels. By integrating *in vitro* and *in vivo* approaches, this study aimed to provide a comprehensive evaluation of the suitability of *E. faecium* as a probiotic candidate for yak health management. Ultimately, the findings are expected to contribute to the development of safe and effective antibiotic alternatives specifically adapted to high-altitude livestock production systems.

## MATERIALS AND METHODS

### Ethical approval

The animal experiments conducted in this study were carried out in accordance with the guidelines for the care and use of laboratory animals and were approved by the Animal Care and Use Committee of Southwest Minzu University, Chengdu, China (Approval No. SWUN-MR2022-0058). The study involved oral administration of *E. faecium* SWUN5732 for the evaluation of probiotic safety and functional effects, including growth performance and intestinal immune response, and did not involve any surgical or invasive procedures.

All animals were housed under controlled environmental conditions with appropriate temperature, relative humidity, and a 12 h light/dark cycle, and had free access to standard feed and water. The animals were monitored daily throughout the experimental period for general health status, behavior, and any signs of adverse effects or toxicity. Particular attention was given to minimizing stress and discomfort during handling and gavage procedures.

At the end of the experimental period, animals were humanely euthanized using carbon dioxide inhalation in accordance with accepted ethical guidelines. Tissue collection was performed promptly following euthanasia to ensure sample integrity and to avoid unnecessary suffering.

The isolation of *E. faecium* SWUN5732 from traditional yak yogurt did not involve live animal experimentation and therefore did not require additional ethical approval.

### Study period and location

This study was conducted from March 2022 to June 2022 at Southwest Minzu University, Chendu, China.

### Instruments and reagents

Instruments and reagents used in this study are presented in Table 1.

**Table 1:** Main instruments and reagents.

Instruments and reagents	Company
Electrophoresis power supply (DYY-3C)	Beijing Liuyi Instrument Factory
Polymerase chain reaction thermal cycler (Veriti™)	Thermo Fisher Scientific Inc
UV-Vis spectrophotometer (UV-6100)	Shanghai INESA Analytical Instrument Co., Ltd
Bio-electrophoresis image analysis system (FR-980A)	Shanghai Furi Technology Co., Ltd.
Microvolume nucleic acid/protein analyzer (NanoDrop)	Thermo Fisher Scientific Inc
MRS agar (M8330)	Solarbio
Gram stain kit	Solarbio
Oxford cup (Inner diameter 6 mm, Outer diameter 8 mm, Height 10 mm)	Chengdu Nuozhou Biotechnology Co., Ltd.
Antibiotic disks	Tianhe Microbial Reagent Co., Ltd.

### Isolation and identification of lactic acid bacteria

*E. faecium* SWUN5732, isolated from traditional fermented yak yogurt, underwent successive streaking on de Man, Rogosa, and Sharpe (MRS) solid medium supplemented with 0.5% calcium carbonate. The plates were incubated under anaerobic conditions at 37°C for 36 h to achieve isolation of individual colonies.

These colonies were then subjected to Gram staining and microscopic examination until a pure staining pattern was achieved. The genomic DNA was extracted using the phenol-chloroform method, and the DNA content and purity were assessed using an ultramicro nucleic acid protein analyzer (Bacterial cells were collected by centrifuging 1 mL of culture), the pellet was resuspended in 500 μL SDS lysis buffer containing proteinase K, and incubated at 60°C for 2 h; cooled to 20°C, an equal volume of phenol-chloroform-isoamyl alcohol (25:24:1) was added, and gently inverted to mix for 10 min, then centrifuged at 4°C, 10,000 × *g* for 15–20 min; the upper aqueous phase was carefully transferred to a new tube, an equal volume of chloroform-isoamyl alcohol (24:1) was added, extracted again, and centrifuged to collect the supernatant; 2 volumes of pre-cooled absolute ethanol were added, incubated at −20°C for 30 min to precipitate DNA, then centrifuged at 4°C, 12,000 × *g* for 15–20 min; the supernatant was discarded, the pellet was washed with 70% ethanol, air dried, and the DNA was dissolved in 50 μL TE buffer, and stored at −20°C.

The PCR product was amplified using 16S rRNA universal primers (27F: 5´-AGAGTTTGATCCTGGCTCAG 3´, 1492R: 5´-GGTTACCTTGTTACGACTT-3´) and the extracted strain DNA as a template (Prepare the PCR amplification system with 12.5 µL 2 × T5 Super PCR Mix, 1 µL each of forward and reverse primers, 2 µL DNA template, and add ddH_2_O to a final volume of 25 µL. PCR program: initial denaturation at 98°C for 3 min; denaturation at 98°C for 10 s; annealing at 54°C for 10 s; extension at 72°C for 15 s; final extension at 72°C for 2 min; 30 cycles total; hold at 4°C. For agarose gel electrophoresis: use 1% agarose gel with 1× TAE as the running buffer to detect PCR products at 120 V, 200 mA for 25 min.). The desired bands were selected, and bidirectional sequencing was performed with the assistance of Tsingke Biotechnology (Chengdu, China). The obtained sequences were analyzed using BLAST analysis in MEGA 11 software (https://www.megasoftware.net), and phylogenetic trees were constructed using the obtained gene sequences.

### Measurement of growth curves

*E. faecium* SWUN5732 was cultured in MRS liquid medium at a concentration of 1% and incubated at a constant temperature of 37°C (Initial concentration of OD600 = 0.2). The optical density at 600 nm (OD600 nm) of the strain was measured at 1 mL intervals every 2 h using a plate reader (e.g., BioTek Synergy H1), and the resulting data were recorded. Report as mean ± standard deviation (SD) from 3 biological replicates.

### Hemolysis test

A single colony of *E. faecium* SWUN5732 was picked and inoculated into hemolysin medium (containing 10% defibrinated rabbit blood). The culture was incubated at 37°C for 24 h. The colony growth status on the blood agar plate was then observed. S. aureus was used as the positive control.

### Acid and bile salt resistance tests

*E. faecium* SWUN5732 was inoculated with MRS broth at 1% and incubated at 37°C for 14 h. The broth was divided into 4 portions. Two portions were adjusted to pH 2.0 and 3.0 with 1 mol/L hydrochloric acid and incubated at 37°C for 3 h. The third bacterial solution was centrifuged at 8,000× g for 10 min. After discarding the supernatant, the organisms were collected and resuspended in MRS broth containing 0.3% bile salts. The suspension was then incubated for 3 h at 37°C. The remaining part of the broth was left untreated and used as a control group. The four groups were diluted in three concentration gradients (10^5^, 10^6^, 10^7^) in a 10-fold gradient and the live bacteria were counted on the plates. The experiment was conducted in three parallel groups and the results were averaged three times. *E. faecium* SWUN5732 was inoculated into MRS liquid medium at the specified percentage and incubated at 37°C for 12 h. The culture was then centrifuged at 8,000× g for 10 min, and the supernatant was discarded to collect the organisms. The bacteria were resuspended in an equal volume of MRS liquid medium containing 0.3% bile salt and incubated at 37°C. At 0 h and 3 h, the bacteria were diluted in a gradient and the appropriate dilution was selected to determine the count of live bacteria. The experiment was performed in three parallel groups, with each group repeated three times. The survival rate was calculated by averaging the results of the three repetitions and using 0 h as a reference. The survival rate formula is shown in Equation 1.

Equation 1: Survival rate (%) = Number of live bacteria in the conditioned treatment/Number of untreated live bacteria × 100%

### Antibiotics sensitivity test

A 10^6^ CFU/mL bacterial suspension was spread onto LB agar plates. The bacterial susceptibility to antibiotics was then determined using the Kirby–Bauer disk diffusion method, and the diameter of the inhibition zones was measured. All experiments were performed in triplicate.

### Determination of common yak-derived diarrhoeal pathogens

The bacteriostatic activity of *E. faecium* SWUN5732 was assessed using the Oxford Cup method, with common yak Enterococcus (Escherichia coli ATCC25922 and Staphylococcus aureus ATCC25923, yak-derived haemolytic Escherichia coli SWUN4569, yak-derived Escherichia coli SWUN4135, yak-derived Salmonella spp. SWUN3710, yak-derived Salmonella spp. SWUN3733, yak-derived Salmonella spp. SWUN3712, yak-derived Salmonella Brittanica SWUN3825, Staphylococcus aureus SWUN4509, yak-derived Escherichia coli emolitico SWUN4566, yak-derived Escherichia coli emolitico SWUN4580, and human colon adenocarcinoma cells (Caco-2) were provided by the Animal Medicine Laboratory of Southwest Minzu University) serving as the indicator strain.

The indicator strain was incubated at 37°C for 12 h, the bacterial solution concentration was diluted to about 10^5^ CFU/mL by continuous 10-fold gradient dilution, and 0.3 mL was applied to the nutrient AGAR (NA) plate. The Oxford Cup was lightly placed on the plate and pressed slightly to ensure there were no gaps between the Oxford Cup and the culture medium. Then, 0.2 mL of the supernatant of the probiotic strain was added and the mixture was allowed to stand for 8 h in the refrigerator at 4°C to diffusion. Afterward, it was placed in a constant temperature incubator at 37°C for 12 h to observe the results. All experiments were performed in triplicate.

### Caco-2 cell adhesion

Caco-2 cells (Maintained in our laboratory) were cultured in sterile cell bottles with 5 mL of complete culture medium and incubated in a cell culture incubator at 37°C with 5% CO2 for 48 h. During passaging, the cells were allowed to adhere to the wall for approximately 80% of the time. Cells between passages 20–40 were used for all experiments to ensure consistent differentiation status.

The Caco-2 cells were digested with ethylenediaminetetraacetic acid (EDTA)-trypsin (0.25% trypsin-EDTA) for 1~2 min in the T25 flask, and the cell concentration was adjusted to 2×10^5^ cells/mL with complete culture medium (Cell viability was assessed using trypan blue exclusion method (>95% viability was required for use). After 12 h of incubation, non-adherent cells were washed with sterile phosphate buffer saline (PBS). Finally, 1 mL of culture medium was added prior to the adhesion assay.

*E. faecium* SWUN5732 was inoculated with MRS liquid medium (1%, v/v) in percentage and incubated at 37°C for 18 h to a stable phase. The sample was centrifuged at 4°C and 8,000 × *g* for 10 min. After discarding the supernatant, the pellet was washed twice with sterile PBS and resuspended in PBS. The concentration was adjusted to 1 × 10^9^ CFU/mL as determined by spectrophotometry (OD600 = 1) and confirmed by plate counting on MRS agar. Different dilutions were prepared from the suspension for viable bacteria counts.

Bacterial suspension (1 mL) was inoculated into each well of a six-well plate (MOI = 100:1, bacteria to cells) and incubated for 12 h. Cells without bacteria were used as control, with three parallel controls conducted for each group and incubated for a period of 2 h at a temperature of 37°C and a CO2 concentration of 5%.

The unattached bacteria were rinsed with sterile PBS, treated with 0.4 mL of trypsin containing EDTA (0.25% trypsin-EDTA, 5 min at 37°C), and subsequently subjected to the addition of 1 mL of culture solution followed by repeated agitation. A gradient dilution of the cell-bacteria mixture was prepared, with varying dilution ratios chosen for the enumeration of viable bacteria. All experiments were performed in triplicate. The adhesion rate formula is shown in Equation 2.

Equation 2: Adhesion rate (%) = (adherent CFU / initial CFU) × 100%

### Safety tests

**Virulence gene assay:** The six common virulence gene primers of Enterococcus were designed and synthesized by Sangon Biotech Co. Ltd., Shanghai, China. These primers were utilized in PCR experiments to amplify the target genes. The bacterial genome extraction procedure was conducted according to 1.4.2, and the specific primer information can be found in Table 2. The PCR amplification was performed using the following thermal cycling program: initial denaturation at 94°C for 5 min; followed by 30 cycles of denaturation at 94°C for 30 s, annealing for 30 s (Table 2), and extension at 72°C for 1 min; with a final extension at 72°C for 7 min, and holding at 4°C.

**Table 2:** Enterococcal virulence gene primer design.

Target gene	Primer sequence (5’~3’)	Annealing temperature /℃	Fragment size/BP
CylA-F	ACTCGGGGATTGATAGGC	55	688
CylA-R	GCTGCTAAAGCTGCGCTT		
Esp-F	AATTGATTCTTTAGCATCTGG	47	510
Esp-R	AGATTCATCTTTGATTCTTGG		
Asa1-F	GCACGCTATTACGAACTATGA	51	375
Asa1-R	TAAGAAAGAACATCACCACGA		
EfaAfm-F	AACAGATCCGCATGAATA	45	735
EfaAfm-R	CATTTCATCATCTGATAGTA		
GelE-F	TATGACAATGCTTTTTGGGAT	48	213
GelE-R	AGATGCACCCGAAATAATATA		
Agg-F	CCAGTAATCAGTCCAGAAACAACC	53	406
Agg-R	TAGCTTTTTTCATTCTTGTGTTTGTT		

CylA = Cytolysin A, Esp = Enterococcal surface protein, asa1 = Aggregation substance of E. faecalis protein 1, EfaAfm = Endocarditis specific antigen of *E. faecium*, GelE = Gelatinase, Agg = Aggregation substance.

**Animal acute toxicity oral test:** The present study was conducted in strict accordance with the requirements of the Animal Care and Use Committee of Southwest Minzu University, Chengdu, China (No. SWUN-MR2022-0058). Twenty male Kun Ming (KM) mice (20 ± 2 g) aged 8 weeks were obtained from Chengdu Dashuo Co. Ltd., Chengdu, China and randomly assigned to two groups (control group and a Treatment group), with 10 mice in each group. The Treatment group received a daily gavage of 1 × 10^9^ CFU/mL bacterial suspension at 0.1 mL/10g body weight, while the control group received the same volume of MRS broth. The mice were housed in cages maintained at a temperature of 22°C ± 2°C, with a relative humidity of 55%–65% and a 12 h light/dark cycle. They were provided with ad libitum access to standard diet food and water. The mice were euthanized using the CO_2_ method after 7 days, and their hearts, livers, spleens, kidneys, and thymus were collected to determine organ indices. The visceral index formula is shown in Equation 3.

Equation 3: Visceral Index (%) = Organs weight (g) / Pre-autopsy weight of mice (g) × 100%

### Determination of the effect of *E. faecium* SWUN5732 on the body weight of mice

Thirty male KM mice (20 ± 2 g) aged 8 weeks were obtained from Chengdu Dashuo Co. Ltd. placed in a cage at 22°C ± 2°C and 55%–65% relative humidity (RH) and under a 12 h/12 h light/dark cycle, and were given free access to standard diet food and water. The mice were randomly assigned to three groups (control, Low-dose, and High-dose groups), with 10 mice in each group. Based on the outcomes of the pre-test, the High-dose group received an administration of 0.2 mL of 5 × 10^9^ CFU of *E. faecium* SWUN5732 (pH = 6), the Low-dose group received an equivalent volume of bacterial solution at a concentration of 5 × 10^6^ CFU (pH = 6), and the control group received an equivalent volume of MRS broth (pH=6), Use 20-gauge gavage needles. Each group of mice was administered the respective dose once daily for a duration of 21 d. The body weights of the mice were measured and recorded on the 1st, 7th, 14th and 21st d.

### Determination of sIgA in the intestinal mucosa

Mice were selected for the experiment using a random sampling method, with three mice chosen from each group at 7, 14, and 21 d. Following euthanasia, the jejunum and ileum mucosa were harvested, and 1 g of intestinal tissue was ground with 1 mL of sterile PBS.

The mixture was then centrifuged at a speed of 6,000× *g* for 15 min. The resulting supernatant was stored at a temperature of −20°C and utilized for subsequent sIgA determination through a double antibody sandwich ELISA, following the instructions provided by the ELISA kit90 (ELISA Kit Catalog Number: JM-02723M1. Jiangsu Jingmei Biotechnology Co. Ltd., China; Standard Curve Method: Linear regression (y = mx + b), R² > 0.99).

### Statistical analysis

All graphs were generated using GraphPad Prism version 8.0.2 (GraphPad Software Inc., San Diego, CA, USA). Statistical analyses were performed with SPSS version 20.0 (Chicago, IL, USA). Data normality was assessed using the Shapiro-Wilk test prior to analysis. One-way analysis of variance was performed to evaluate significant differences among groups, followed by Tukey’s post-hoc test for multiple comparisons. Data are expressed as the mean ± SD with n = 3 for each group. Statistical significance was set at p < 0.05. Bars with different lowercase letters within the same group indicate a significant difference (p < 0.05).

### Bacterial species collection information

The sequence of *E. faecium* SWUN5732 was collected to the China Center for Type Culture Collection with the number of CCTCC NO: M 20221212.

## RESULTS

### Morphological characteristics of the colony, 16S rRNA gene sequence analysis, and phylogenetic tree construction

**Figure 1:**
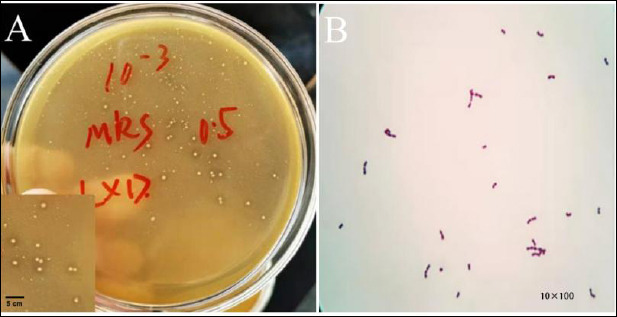
Isolation and identification of SWUN5732 strain. (A) Colony morphology and calcium dissolving circle. (B) Microscopic examination (10× and 100×).

The colony morphology exhibited elevated white circular colonies featuring clearly visible calcium soluble circles that developed on the surface of the calcium carbonate-containing medium (Figure 1-A). Under microscopic examination, the colonies displayed a round or oval shape, consisting of either individual or paired Gram-positive cocci (Figure 1-B).

**Figure 2:**
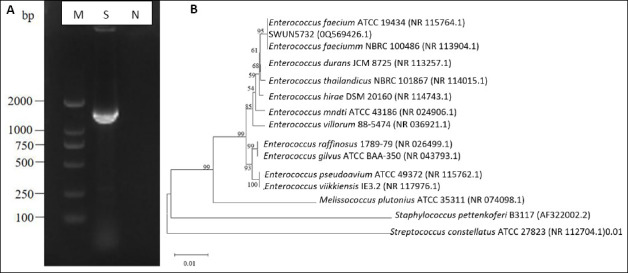
(A) Electrophoresis identification of PCR products amplifying Lactobacillus 16S rRNA sequence. M: DL2000 bp; S: SWUN5732 16S rRNA PCR product; N: Negative control. (B) SWUN5732 phylogenetic tree.

The agar gel electrophoresis results revealed that the amplification fragment of the *16S rRNA* gene was approximately 1500 bp (Figure 2-A), which aligns with the anticipated target fragment. To construct a phylogenetic tree, the *E. faecium* SWUN5732 sequence was combined with various species of Gram-positive cocci sourced from GenBank. The results are shown in Figure 2-B. The strain exhibits 99% identity to *E. faecium* ATCC19434.

### Determination of growth curves

**Figure 3:**
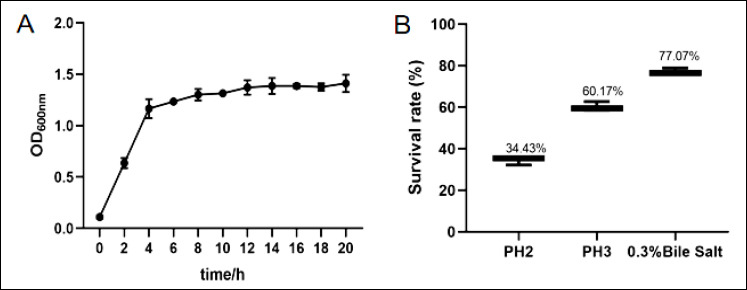
(A) Growth curve of *E. faecium* SWUN5732; (B) Results of acid and bile salt tolerance tests.

The horizontal coordinate for the growth curves was selected as the incubation time, while the vertical coordinate was designated as OD600 nm. Strain *E. faecium* SWUN5732 exhibited a period of rapid growth between 2 and 6 h, followed by a gradual transition into a plateau phase after 6 h, showing a flat trend (Figure 3 A).

### Acid- and bile salt-tolerant

The strain *E. faecium* SWUN5732 demonstrated differential survival rates in relation to distinct pH levels. More precisely, at pH 2.0, the survival rate of *E. faecium* SWUN5732 was determined to be 34.43%, whereas at pH 3.0, the survival rate escalated to 60.17%. Furthermore, upon exposure to 0.3% bile salt, the survival rate of *E. faecium* SWUN5732 was quantified at 77.07% (Figure 3 B).

### Hemolysis test

**Figure 4:**
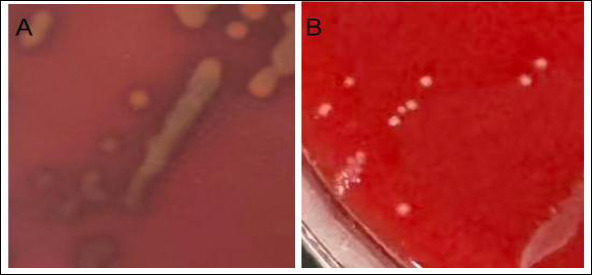
Hemolysis test results. (A) *Staphylococcus aureus*; (B) *Enterococcus faecium* SWUN5732.

Using *Staphylococcus aureus* as the positive control (Figure 4 A), *E. faecium* SWUN5732 showed no hemolytic activity (Figure 4 B).

### Determination of common yak-derived diarrhoeal pathogens

**Figure 5:**
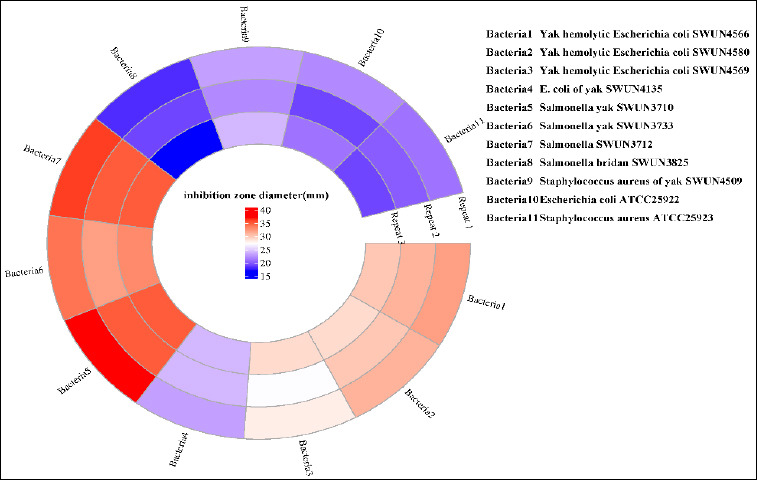
Diameter of the zone of inhibition.

*E. faecium* SWUN5732 produced a product that inhibited pathogenic bacteria of yak origin. It was more effective against *E. coli* SWUN4566 and SWUN4580 than SWUN4569 and SWUN4135. It showed stronger activity against *Salmonella* SWUN3710, SWUN3733, and SWUN3712 than SWUN3825. The specific bacteriostatic effect is shown in Figure 5 and Table 3.

**Table 3:** Inhibition effect of SWUN5732 on intestinal pathogens of yak origin.

Indicating strain	Bacteriostatic zone diameter (mm)
Yak hemolytic *Escherichia coli* SWUN4566	32 ± 1
Yak hemolytic *Escherichia coli* SWUN4580	31 ± 1.5
Yak hemolytic *Escherichia coli* SWUN4569	28 ± 0.75
*Escherichia coli* of yak SWUN4135	23 ± 1.5
*Salmonella* yak SWUN3710	37 ± 0.6
*Salmonella* yak SWUN3733	34 ± 1.2
Salmonella SWUN3712	36 ± 1.6
*Salmonella* SWUN3825	18 ± 0.5
*Staphylococcus aureus* of yak SWUN4509	23 ± 0.7
*Escherichia coli* ATCC25922	22 ± 1.2
*Staphylococcus aureus* ATCC25923	21 ± 0.6

### Antibiotic sensitivity test

*E. faecium* SWUN5732’s antibiotic susceptibility test results are shown in Table 4. *E. faecium* SWUN5732 is susceptible to Ampicillin, Gentamicin, Tetracycline, Minocycline, Ciprofloxacin, Norfloxacin, Chloramphenicol, and Vancomycin.

**Table 4:** Antibiotic susceptibility test results of *Enterococcus faecium* SWUN5732.

Antibiotic name	Susceptible (S)	Intermediate (I)	Resistant (R)	Bactericidal zone diameter (mm)
Ampicillin (10 pg per tablet)	≥17	---	≤16	25 (S)
Gentamicin (10 µg per tablet)	≥15	13 ≤ I ≤ 14	≤12	16 (S)
Tetracycline (30 µg per tablet)	≥19	15 ≤ I ≤ 18	≤14	22 (S)
Minocycline (30 mg per tablet)	≥19	15 ≤ I ≤ 18	≤14	25 (S)
Ciprofloxacin (5 µg per tablet)	≥21	16 ≤ I ≤ 20	≤15	22 (S)
Norfloxacin (10 pg per tablet)	≥17	13 ≤ I ≤ 16	≤12	22 (S)
Chloramphenicol (30 µg per tablet)	≥18	13 ≤ I ≤ 17	≤12	22 (S)
Vancomycin (30 μg per tablet)	≥17 mm	---	<17	19 (S)

### Adherence to Caco-2 cells

By comparing the number of colonies before and after adhesion in three parallel groups of samples, the adhesion rate of *E. faecium* SWUN5732 to Caco-2 cells was obtained to be 28.15%–30.04%. The data are shown in Table 5.

**Table 5:** Cell adhesion rate results.

Group	Initial colony count (CFU/mL)	The number of colonies recovered after adhesion (CFU/mL)	Adhesion rate (%)
Repetition Group 1	5.2 × 10⁸	1.46 × 10⁸	28.15
Repetition Group 2	5.2 × 10⁸	1.52 × 10⁸	29.23
Repetition Group 3	5.2 × 10⁸	1.56 × 10⁸	30.04
Average ± standard deviation	---	---	29.14 ± 0.96

### Safety determination

**Figure 6:**
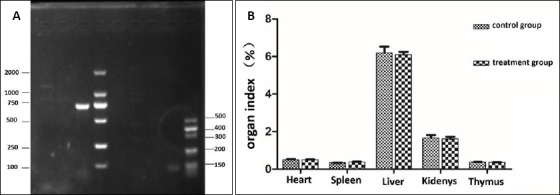
(A) Enterococcal virulence gene results. DL, M: Marker; AG: Agg; GE: GelE; ESP: Esp; ASA: asa1; EF: efaAfm; CY: cylA: PCR product; N: negative control. (B) Results of organ index.

After identification by PCR, only endocarditis antigen (*efaAfm*) among the common virulence genes matched the target bands. The strain was tentatively identified as carrying the *efaAfm* virulence gene. No bands were observed for other tested virulence genes (including *cylA*, *esp*, etc.), suggesting that these virulence factors are not present in this isolate (Figure 6 A).

In the investigation of acute toxicity of an oral bacterial solution in animals, it was observed that none of the mice experienced mortality, and no noteworthy abnormalities were detected. Dissection revealed no significant differences in organ color and size in the blank mice compared to the experimental group and no lesions. The organ weights of the mice were measured, and there was no significant difference in the organ index between the Treatment group and the broth control group (p > 0.05) (Figure 6 B).

### Measurement of daily weight gain in mice

Following a 7 d intragastric administration of *E. faecium* SWUN5732 bacterial solution, a notable disparity in body weight was observed between the 5 × 10^9^ CFU/mL group and the control group (p < 0.05), indicating a positive association with the quantity of viable bacteria administered intragastrically. The growth rate peaked on the 14th d, with a significant distinction observed between the 5 × 10^9^ CFU/mL group and the control group (p < 0.01) (Table 6).

**Table 6:** Weight gain experiment results of SWUN5732 in mice (body weight/g).

Gastric filling volume (CFU/mL)	Day 1	Day 7	Day 14	Day 21
control	31.46 ± 1.99	35.69 ± 2.19	41.95 ± 3.31	42.97 ± 0.92
Low-dose	33.08 ± 1.84	36.79 ± 2.96	42.94 ± 3.45	43.06 ± 2.79
High-dose	32.20 ± 1.31	37.55 ± 2.99*	44.13 ± 4.87**	45.16 ± 4.16**

Values are means ± SEM,

* indicates significant difference (p < 0.05),

** indicates highly significant difference (*p* < 0.01).

### Determination of sIgA in intestinal mucosa

**Figure 7:**
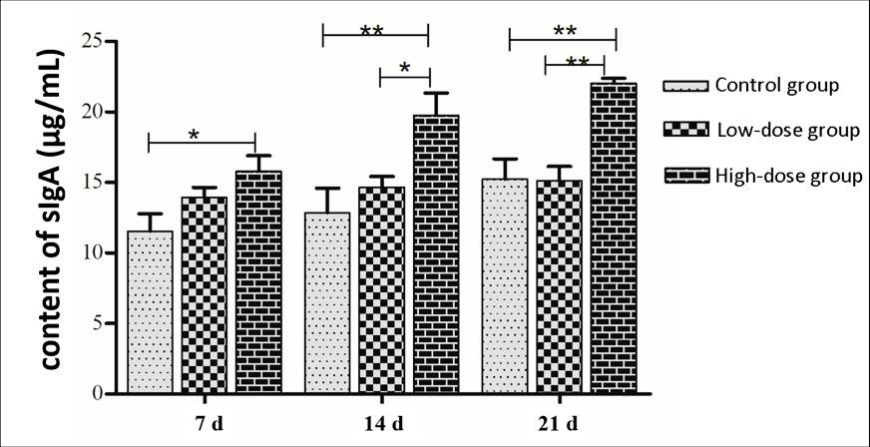
Intestinal sIgA secretion.* indicates significant difference (p < 0.05), ** indicates highly significant difference (p < 0.01).

After a period of 7 d of administering the *E. faecium* SWUN5732 strain via gavage, the secretion of intestinal sIgA was observed (Figure 7). A noticeable trend of increased secretion of sIgA in mice was observed in the High-dose group when compared to the control group. By 14 d, this difference became highly significant (p < 0.01) in the High-dose group compared to the control group, and significant (p < 0.05) when compared to the Low-dose group. After 21 d of gavage, the difference remained highly significant (p < 0.01) in the High-dose group when compared to both the control and Low-dose groups.

## DISCUSSION

### Probiotic potential and identification of E. faecium SWUN5732

The escalating prevalence of antimicrobial resistance has necessitated the development of safe and effective alternatives to antibiotics in animal production. Probiotics, particularly lactic acid bacteria, have emerged as promising candidates due to their multifaceted beneficial effects on host health [18]. In this study, *E. faecium* was isolated from yak yogurt and subsequently identified using *16S rRNA* and evolutionary tree analysis. The findings of this study indicate that *E. faecium* SWUN5732, derived from yak yogurt, exhibited a rapid growth rate, prolonged plateau maintenance period, resilience to the gastrointestinal environment, adherence to the intestinal tract, stimulation of sIgA secretion, as well as the ability to inhibit yak pathogens, collectively supporting its potential application as a microecological agent in yak farming systems.

### Safety evaluation and virulence gene assessment

Safety assessment remains paramount for enterococcal probiotics given the documented pathogenic potential of certain strains, particularly *E. faecalis* and hospital-associated *E. faecium* lineages [19, 20]. Our virulence gene screening revealed the presence of *efaAfm* but absence of major determinants including *cylA*, *esp*, *asa1*, *gelE*, and *agg* [20–22]. While the presence of *efaAfm* warrants consideration, previous studies have demonstrated that this gene alone does not confer pathogenicity and is commonly detected in commensal enterococci without association to clinical disease [23]. The absence of acute toxicity in murine models, normal organ histopathology, and comparable organ indices between treated and control groups collectively support the safety profile of SWUN5732 for oral administration at probiotic doses.

### Adaptation to extreme environments and growth characteristics

Traditional fermented dairy products represent valuable reservoirs for isolating probiotic strains with unique ecological adaptations. The Tibetan Plateau presents extreme environmental conditions, including high-altitude, hypoxia, intense ultraviolet radiation, and low temperatures, which impose selective pressures on indigenous microorganisms [24, 25]. Consequently, microorganisms derived from such environments often possess distinctive physiological characteristics that may confer competitive advantages for applications in high-altitude livestock systems. *E. faecium* SWUN5732 demonstrated rapid growth during the exponential phase (2–6 h) and maintained prolonged stationary phase stability, suggesting efficient colonization potential and sustained metabolic activity in the host gastrointestinal tract.

### Gastrointestinal tolerance and probiotic viability

Probiotics that exhibit favorable performance must possess the ability to endure the acidic conditions and presence of bile salts within the gastrointestinal tract [26]. The gastric environment presents significant challenges, with pH values frequently dropping below 3.0 during fasting states, while bile salts in the small intestine can reach concentrations of 0.3–3.0 g/L [27]. *E. faecium* SWUN5732 exhibited survival rates of 34.43% at pH 2.0, 60.17% at pH 3.0, and 77.07% in 0.3% bile salts. These tolerance profiles compare favorably with previously reported probiotic enterococci and are physiologically relevant for passage through the monogastric stomach and proximal small intestine. The substantial survival at pH 3.0, in particular, indicates that a significant proportion of ingested bacteria would remain viable during gastric transit, whereas the excellent bile salt tolerance suggests robust adaptation to the intestinal environment where probiotic effects are primarily mediated.

### Antimicrobial activity against yak-derived pathogens

The antimicrobial efficacy of *E. faecium* SWUN5732 against pathogenic bacteria of yak origin constitutes a significant finding with practical implications for diarrheal disease management. The strain demonstrated pronounced inhibitory effects against multiple *E. coli* and *Salmonella* isolates, with inhibition zone diameters ranging from 18 to 37 mm. Notably, the strain exhibited differential inhibitory potency among pathogenic isolates, with stronger activity against hemolytic *E. coli* strains and certain *Salmonella* serovars compared to others. This variation may reflect differences in cell wall composition, membrane permeability, or susceptibility to the specific antimicrobial compounds produced by SWUN5732 [28]. The production of bacteriocin-like inhibitory substances, organic acids, and hydrogen peroxide by enterococci has been well-documented [19, 29]; however, the precise nature of the antimicrobial factors produced by SWUN5732 warrants further characterization through purification and mass spectrometry-based identification.

### Adhesion ability and intestinal colonization

Intestinal adhesion represents a prerequisite for effective probiotic colonization and competitive exclusion of pathogens [30]. The adhesion rate of *E. faecium* SWUN5732 to Caco-2 cells (28.15–30.04%) falls within the range reported for established probiotic enterococci and exceeds the threshold generally considered indicative of meaningful host interaction. This adhesion capability likely involves specific molecular mechanisms, including surface proteins and extracellular polysaccharides that mediate host–microbe interactions [31, 32]. The ability to adhere to intestinal epithelial cells enables probiotics to compete with enteric pathogens for receptor sites, thereby preventing pathogen attachment and subsequent infection, a mechanism particularly relevant for controlling enteric diseases in yaks maintained under intensive farming conditions.

### Immunomodulatory effects and sIgA response

The immunomodulatory capacity of probiotics represents an increasingly recognized mechanism underlying their health-promoting effects [33, 34]. Our study demonstrated that oral administration of *E. faecium* SWUN5732 significantly elevated intestinal sIgA concentrations in a dose-dependent manner, with highly significant effects observed from day 14 onwards. Secretory IgA constitutes the predominant immunoglobulin at mucosal surfaces and serves as the first line of defense against enteric pathogens through immune exclusion, neutralization of toxins, and maintenance of microbiota homeostasis. The enhanced sIgA secretion observed in the high-dose group suggests activation of the gut-associated lymphoid tissue and stimulation of B-cell differentiation into IgA-producing plasma cells, a mechanism that may provide systemic benefits beyond the gastrointestinal tract through the common mucosal immune system.

### Growth performance and physiological benefits

The concomitant enhancement of body weight gain in treated mice further supports the physiological relevance of SWUN5732 supplementation. The average weight increase of 2.19 g in the high-dose group relative to controls, particularly evident during the rapid growth phase (days 7–14), likely reflects improved nutrient utilization efficiency and reduced metabolic costs associated with immune activation and pathogen defense [35]. This growth-promoting effect, achieved without antibiotic exposure, aligns with the strategic objective of reducing antimicrobial use in livestock production while maintaining animal performance [15, 36–38].

### Study limitations and future perspectives

Several limitations of this study should be acknowledged. The murine model, while informative for preliminary safety and efficacy assessment, does not fully replicate the anatomical and physiological characteristics of the yak digestive system, particularly the rumen and its associated microbial ecosystem. And, the duration of the intervention (21 d) provides limited insight into long-term colonization dynamics and sustained immunomodulatory effects. The antimicrobial activity was assessed using *in vitro* diffusion assays, which may not accurately reflect *in vivo* efficacy within the complex gut environment. The molecular mechanisms underlying sIgA induction and the specific bacterial components responsible for immune stimulation remain to be elucidated. Future investigations should address these gaps through rumen-fistulated yak studies, longitudinal colonization monitoring, transcriptomic analysis of immune responses, and metabolomic profiling of bacterial supernatants.

## CONCLUSION

The present study demonstrated that *E. faecium* SWUN5732, isolated from traditional yak yogurt, possesses multiple probiotic characteristics that collectively support its functional applicability. The strain exhibited rapid growth kinetics, strong tolerance to acidic and bile salt conditions, effective adhesion to intestinal epithelial cells, and pronounced antimicrobial activity against yak-derived pathogens, including *Escherichia coli* and *Salmonella* spp. In addition, *in vivo* evaluation revealed enhanced intestinal sIgA secretion and improved body weight gain in treated mice, indicating significant immunomodulatory and growth-promoting effects.

Safety assessment confirmed the absence of major virulence determinants except *efaAfm*, which alone is not associated with pathogenicity, and no adverse effects were observed in acute toxicity studies. These findings indicate that *E. faecium* SWUN5732 is safe for oral administration at probiotic doses and suitable for further development as a functional microbial agent.

From a practical perspective, the ability of *E. faecium* SWUN5732 to inhibit enteric pathogens, enhance mucosal immunity, and improve growth performance suggests its potential application as a natural alternative to antibiotics in yak production systems. Its origin from traditional yak yogurt and adaptation to high-altitude environmental conditions further support its ecological compatibility and potential effectiveness in plateau livestock farming.

A major strength of this study lies in the comprehensive evaluation of probiotic potential through both *in vitro* and *in vivo* approaches, combined with the use of yak-derived pathogenic strains, which enhances the applicability of the findings.

Overall, *E. faecium* SWUN5732 represents a promising probiotic candidate with multifunctional benefits, including antimicrobial activity, intestinal colonization, immunomodulation, and growth promotion. Its characteristics support its potential as a sustainable alternative to antibiotics in yak husbandry, although further validation in the target species is required to confirm its efficacy and feasibility under field conditions.

## DATA AVAILABILITY

The supplementary data can be made available from the corresponding author upon request.

## AUTHORS’ CONTRIBUTIONS

MY: Writing–original draft; Formal analysis; Investigation; Visualization. XBL: Writing–original draft; Methodology; Validation; Resources. LJR: Conceptualization; Supervision; Project administration; Funding acquisition. XR: Data curation; Software; Formal analysis; Investigation. XDL: Methodology; Investigation; Resources; Validation. DCC: Methodology; Writing – review and editing; Funding acquisition; Supervision; Project administration. FLY: Software; Data curation; Formal analysis; Visualization. JF: Writing – review and editing; Conceptualization; Supervision; Resources. All authors read and approved the final manuscript.
